# Effects of afforestation on Technosol properties in reclaimed hard coal deep mining spoil heaps

**DOI:** 10.1038/s41598-026-37992-z

**Published:** 2026-02-02

**Authors:** Marcin Pietrzykowski, Amisalu Milkias Misebo, Bartłomiej Woś, Marek Pająk

**Affiliations:** 1 Faculty of Forestry, Department of Ecological Engineering and Forest Hydrology, University of Agriculture in Krakow, Krakow, Poland; 2 Department of Environmental Science, Wolaita Sodo University, Wolaita Sodo, Ethiopia

**Keywords:** Carbon, Restoration, Plantation, Post-mining, Soil, Succession, Biogeochemistry, Ecology, Ecology, Environmental sciences

## Abstract

Mining for fossil fuels and minerals generates spoil heaps and open pits, which have significant environmental impacts in addition to their economic contributions. Afforestation of these disturbed areas can improve soil properties, thereby increasing the functionality and resilience of terrestrial ecosystems. However, the extent of changes in soil properties following afforestation varies depending on the methods used for tree introduction. There is a need for knowledge on the effects of afforestation on soil properties, especially in post-mining Techonosols. Therefore, the objective of this research is to evaluate the effects of three afforestation methods, succession on barren spoil top (SBT), succession on reclaimed topsoil (STS), and plantation on reclaimed topsoil (PTS), on soil properties in a coal post-mining site. Soil samples were collected from 30 randomly established plots (10 × 10 m) for physical and chemical analyses, focusing on the upper layer (0–10 cm depth). The collected samples were analyzed for soil texture, bulk density (BD), porosity, air capacity, capillary water capacity (CWC), moisture content (MC), exchangeable base cations (Ca^2^⁺, Mg^2^⁺, K⁺, and Na⁺), total organic carbon (SOCt), SOC fractions, total nitrogen (Nt), and total sulfur (St). The results showed that PTS had significantly higher CWC, Nt, Ca^2^⁺, K⁺, occluded light fraction of carbon (ColF), and mineral-associated carbon fraction (CMAF) compared to SBT. These improvements highlight the effectiveness of active reclamation in enhancing soil structure stability, nutrient retention, and long-term carbon stabilization, critical elements for post-mining ecosystem restoration. In contrast, SBT had greater porosity, Mg^2^⁺, and free light fraction of carbon (CflF) than STS. In addition, SBT had greater St compared to STS and PTS. This indicates that both natural succession and active restoration contribute to soil change through different mechanisms. Therefore, the choice between afforestation strategies should depend on factors such as restoration objectives, topsoil availability, and resource constraints, as active restoration is labor-intensive and costly.

## Introduction

The afforestation of post-mining sites is a critical ecological restoration strategy aimed at rehabilitating degraded landscapes and improving soil health^1,2^. Mining activities lead to the removal of vegetation and topsoil, resulting in severe degradation of soil quality. Mining operations often result in significant alterations to soil properties, including loss of organic matter, compaction, and changes in nutrient availability^3,4^. These mining activities result in post-mining Technosols characterized by massive overburden dumps, pollution, CO_₂_ emissions, lower soil organic carbon (SOC), and nutrient-poor soils, which hinder ecosystem development^5,6^. Additionally, mining can cause off-site pollution through acid mine drainage^7,8^.

Despite these challenges, restoration efforts through afforestation are essential for restoring these degraded landscapes. Effective restoration methods improve soil properties such as organic matter content, microbial activity, soil moisture, and cation exchange capacity, which are critical for vegetation growth and ecosystem recovery^9–11^. Key soil parameters for successful restoration include soil organic carbon (SOC), total nitrogen (TN), total sulfur (St), exchangeable base cations including Ca^2^⁺, Mg^2^⁺, K⁺, and Na⁺, bulk density, water-holding capacity, air capacity, and porosity^12,13^.

Afforestation has been shown to significantly enhance SOC levels in post-mining Technosols. As vegetation establishes itself, organic matter inputs from leaf litter, root biomass, and decaying plant material contribute to increased SOC accumulation^14,15^. Vegetation types used in restoration significantly influence carbon cycling by storing carbon in biomass, SOM, and litter. Different vegetation and reclamation methods affect SOC and TN storage due to variations in organic matter input, decomposition rates, and distribution at various soil depths^16–18^. Vindušková and Frouz^19^ found lower soil carbon storage in coniferous forests compared to grasslands and deciduous forests on reclaimed mining sites in the Northern Hemisphere. Reclamation with topsoil results in significantly higher SOC accumulation compared to barren spoil top, underscoring the importance of reclamation for enhancing soil quality and carbon storage^20,21^^.^

In addition to increasing SOC, afforestation positively influences other soil nutrients essential for plant growth. Following afforestation, studies have reported improvements in key nutrients such as nitrogen (N), phosphorus (P), and potassium (K) within the soil profile ^22,23^. The presence of vegetation facilitates nutrient cycling processes through root exudation and microbial activity, which enhances nutrient availability for subsequent plant growth^2,24^. For example, a study conducted on reclaimed mine lands found that afforested areas exhibited significantly higher nitrogen levels compared to unplanted areas^25^. The increase in nitrogen content is particularly important for promoting healthy plant growth and establishing a self-sustaining ecosystem^26^. Furthermore, the microbial community composition shifts favorably with the establishment of vegetation; studies indicate that afforested soils have higher microbial biomass and activity compared to bare or minimally vegetated soils^27,28^.

Afforestation also contributes to improvements in soil structure and water retention capacity. The development of root systems from trees helps break up compacted soils, enhancing porosity and aeration^29^. Improved soil structure facilitates better water infiltration and retention, which is crucial for sustaining plant growth in post-mining Technosols where water availability may be limited^12,30^. Research by Singh et al.^31^ has shown that reclaimed mine soils under tree cover exhibit increased water holding capacity compared to bare soils. This is particularly beneficial for mitigating erosion risks associated with heavy rainfall events common in many mining regions. The establishment of vegetation not only stabilizes the soil but also reduces surface runoff, promoting better water conservation practices^32^.

Afforestation of post-mining Technosols is primarily executed through two approaches: spontaneous succession and the deliberate planting of selected tree species in reclaimed areas. These methods can have varying impacts on soil properties, influenced by factors such as soil composition, climate, and the specific tree species involved. Despite the recognized importance of these afforestation strategies, there is a notable lack of comprehensive and cohesive studies examining how different afforestation techniques affect Technosol characteristics in hard coal post-mining sites. Therefore, this study aims to systematically evaluate the effects of various afforestation methods specifically, succession on barren spoil top, succession on reclaimed topsoil, and plantation on reclaimed topsoil on Technosol properties in hard coal post-mining sites. By addressing this gap in the literature, the research seeks to contribute valuable insights into optimizing reclamation practices and enhancing ecological restoration efforts in disturbed landscapes.

## Material and methods

### Study site

The study site was the Sośnica hard coal post-mining spoil heap, situated in the Gliwice and Zabrze regions of the Upper Silesian Coal Basin in southern Poland (50°16′22" N, 18°44′43" E). This area experiences a temperate climate characterized by an average annual temperature of 8.5 °C and mean annual precipitation of 727 mm. The site has a historical association with hard coal mining that spans over 250 years and covers approximately 170 hectares, with elevations exceeding 30 m. The spoil heap primarily consists of Carboniferous rocks, including shale, sandstone, and conglomerates. These substrates are noted for their poor water retention capabilities, a rapidly drying surface layer, low soil organic matter (SOM) content, limited nutrient availability, high salinity levels, and significant geogenic (fossil) carbon content^29,33^. The spoil heap exhibits anthropogenic microrelief with heterogeneous topography, where slopes transition from steep (unstable) to gentle (revegetated), reflecting differential weathering and stabilization dynamics.

Reclamation efforts commenced with the grading and leveling of the spoil heaps, followed by the application of approximately 50 cm of topsoil in selected areas^34^. Reclaimed sites now support diverse vegetation, including pioneer forbs (e.g., *Lupinus polyphyllus, Melilotus alba*), grasses (e.g., *Festuca rubra, Arrhenatherum elatius*), and tree species^29^. Over 20–25 years, natural succession has enhanced vegetation establishment across both reclaimed and unreclaimed areas. The current stand is dominated by *Robinia pseudoacacia*, *Betula pendula, Populus tremula, Pinus sylvestris, Alnus glutinosa, Salix alba*, and *Populus hybrids*.

### Soil sampling and laboratory analyses

**Fig. 1:**
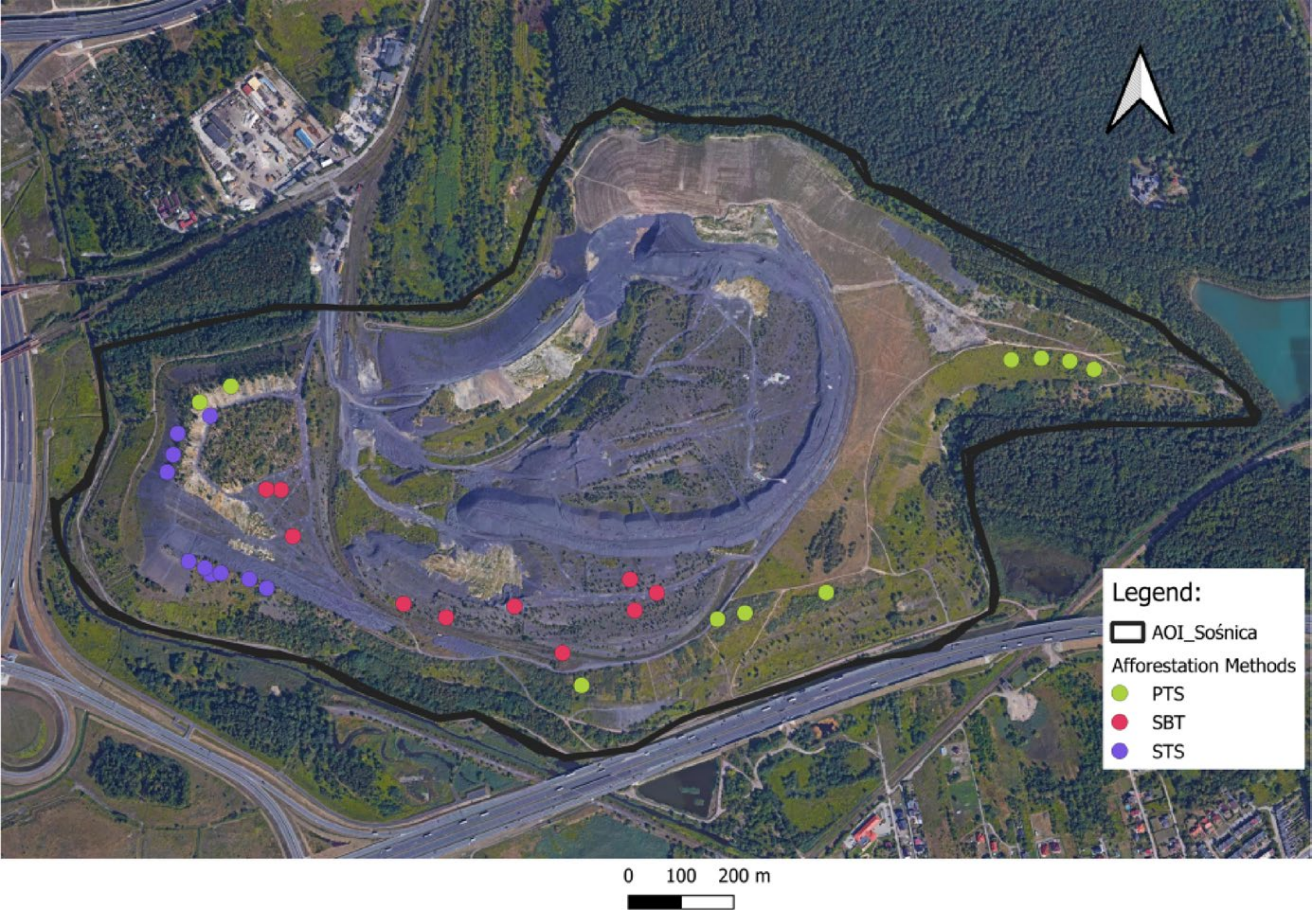
Research plots in the area of interest (AOI) of the Sośnica hard coal post-mining spoil heap. *(The satellite basemap imagery is * *© 2024 Google. The map was generated by the authors using QGIS geographic information system software (version 3.44; QGIS Development Team, 2024; URL: *http://www.qgis.org*))*.

**Fig. 2:**
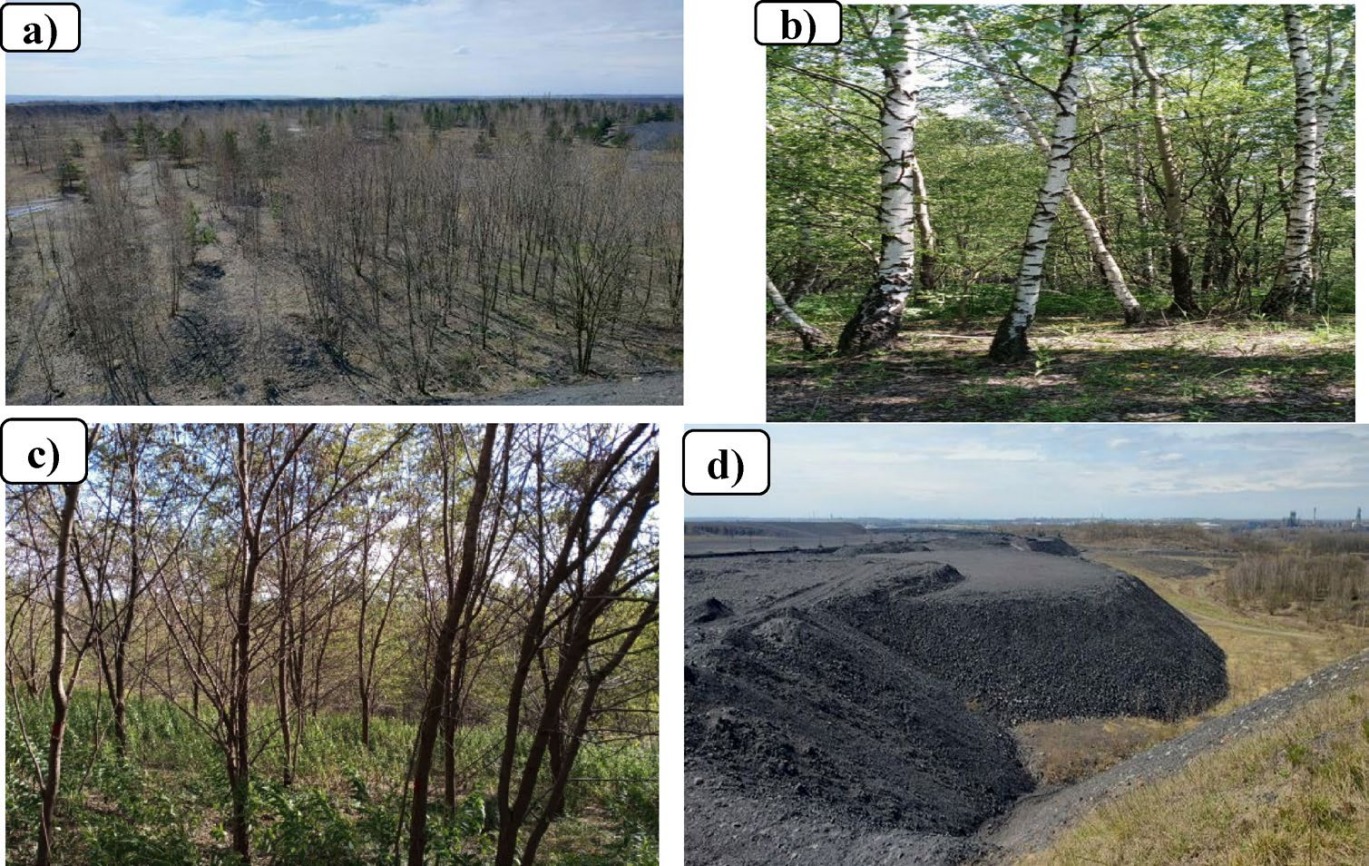
Picture of vegetation developed on the study mining heap; a) succession on barren spoil top b) planation on reclaimed topsoil c) succession on reclaimed topsoil d) barren spoil top without vegetation (photo by A.M. Misebo).

Soil samples were collected from sites being restored with different tree species using different afforestation methods in October 2021 (Table 1 & Fig. 1). Research plots for different treatments were established on similar slope and aspect conditions to minimize the influence of physical setting on soil properties. A total of 30 research plots, with ten replications of each variant of 10 × 10 m, were randomly established across the identified experimental patches on the spoil heap (Figs. 1 & 2). Moreover, ten research plots were established on barren rock areas to account for the influence of geogenic carbon content in the calculation of soil organic carbon (SOC) under succession on barren spoil top. Composite topsoil samples (0–10 cm depth) were collected from five subsampling points (four corners and plot center) per plot. Due to limited pedogenesis and substantial geogenic carbon in unweathered overburden/rock fragments, sampling was confined to the uppermost topsoil horizon. Soil bulk density (BD) was determined by collecting undisturbed core samples from the center of each plot using stainless steel cylinders (100 cm^3^ volume; 5.0 cm diameter × 5.1 cm height) to preserve intact soil structure. The collected composite mineral soil samples were air-dried, sieved through a 2 mm mesh, and subjected to analyses for selected physicochemical properties.

**Table 1:** Description of site categories

	Afforestation		
	SBT	STS	PTS
Reclamation method	–	Topsoiling	Topsoiling
Vegetation management	Natural succession	Natural succession	Plantation of selected trees

SBT, succession on barren spoil top; STS, succession on reclaimed topsoil; PTS, plantation on reclaimed topsoil.

To determine bulk density (BD), porosity, air capacity, capillary water capacity (CWC), and moisture content (MC), undisturbed soil samples were first weighed to determine their initial water content. The samples were then saturated by capillarity for 48 h and weighed after allowing excess water to drain until gravitational drainage ceased. The soil samples were then oven dried at 105 °C for 48 h and weighed again. Bulk density (BD, g cm⁻^3^) was calculated as the ratio of the oven-dried soil mass to the volume of the sampling cylinder, as described by Sumner^35^.

1 $$Porosity (\%)=\frac{\text{Volume of pores}}{\text{Total volume of soil}}*100$$

The pore volume of the undisturbed soil samples was determined directly using the core saturation method^36^. This method relies on the volumetric displacement principle upon full saturation of a dried sample of known total volume.

2 $$MC=\frac{\mathrm{Mw}-\mathrm{Md}}{\mathrm{Md}}*100$$

where:

- MC = Moisture Content (%)
- Mw = Mass of the moist soil before saturation (grams)
- Md = Mass of the oven-dry soil (grams)

                                                                         

3 $$\text{Air Capacity}(\%)=\mathrm{Porosity}(\%)-\text{Water Content}(\%)$$

4 $$CWC=\frac{\mathrm{Mwet}-\mathrm{Mdry}}{\mathrm{Mdry}}*100$$

where:

- CWC = Capillary Water Holding Capacity (%)
- M*wet* = Mass of the soil sample after saturation (grams)
- M*dry* = Mass of the oven-dry soil sample (grams)

Soil texture was evaluated using a Fritsch GmbH Laser Particle Sizer (ANALYSETTE 22). Total organic carbon (SOCt), total nitrogen (Nt) and total sulfur (St) concentrations were determined using a LECO TruMac® CNS analyzer. The pH of the samples was measured in a 1 M KCl solution (with a soil-to-liquid ratio of 1:5, w/v) using a digital pH meter at 20 °C. Exchangeable base cations, including Ca^2^⁺, Mg^2^⁺, K⁺, and Na⁺, were analyzed using a Thermo Scientific™ iCAP™ 6000 Series ICP-OES instrument with 1 mol/L NH4Ac.

The SOC content during succession on barren overburden without topsoil was determined by subtracting the carbon content of the recent unvegetated overburden from that of the soil under succession, assuming similar initial overburden conditions to minimize the influence of geogenic coal carbon, following methods used in previous studies^9,37^.

For soil organic matter (SOM) fractionation, 20 g of sieved soil (< 2 mm) was placed in a 200 ml test tube. Then, 90 ml of sodium polytungstate (SPT, density 1.8 g cm^-3^) was added, followed by shaking for 1 min and centrifugation for 30 min. The free light fraction (fLF) was extracted with a pipette and collected on a glass fiber filter. The remaining soil in the centrifuge tubes was treated with an additional 90 ml of SPT and sonicated at 60 W for 200 s to break up aggregates, after which the occluded light fraction (oLF) was collected on another glass fiber filter. The fraction remaining at the bottom of the tube was classified as the mineral-associated fraction (MAF). After drying, the weights of the fractionated samples were recorded, and the carbon content of each fraction (fLF, oLF, and MAF) was analyzed using a LECO TruMac® CNS analyzer as described by von Lützow et al.^38^.

### Statistical analysis

Descriptive analysis was employed to evaluate selected general soil properties in the study area. Prior to analysis, the datasets were tested for normality using the Kolmogorov–Smirnov test. One-way analysis of variance (ANOVA) was used to investigate the effects of different afforestation methods on soil properties. Means, standard deviations, and Tukey’s honestly significant difference (HSD) tests were calculated. Statistica 13.3 (StatSoft, Inc., 2014) and R software were used for statistical analyses. Means were considered significantly different at p < 0.05. Correlations between soil nutrients and CfLF, CoLF, CMAF, and SOCt were described using a Pearson correlation matrix (p < 0.05).

## Results

### Basic soil properties

Soil under succession on a barren spoil top (SBT) had a significantly higher sand content than soil under succession on reclaimed topsoil (STS). However, it also had significantly lower silt content and lower pH values than both STS and plantation on reclaimed topsoil (PTS). In addition, bulk density (BD) was significantly higher under STS than under SBT and PTS (Table 2).

**Table 2:** Basic soil properties.

Basic soil properties	Afforestation		
	SBT	STS	PTS
Sand (%)	66 ± 5.21^a1^	41 ± 2.70^b^	51 ± 14.93^ab^
Silt (%)	26 ± 3.35^b^	48 ± 3.74^a^	41 ± 13.30^a^
Clay (%)	8 ± 1.98^a^	11 ± 3.74^a^	8 ± 1.98^a^
pH	4.66 ± 0.44^b^	6.62 ± 0.95^a^	7.29 ± 0.31^a^
BD (Mg m^-3^)	1.21 ± 0.12^b^	1.59 ± 0.11^a^	1.31 ± 0.17^b^

^1^different letters indicate significant differences at p < 0.05 along the row under different afforestation methods

SBR, succession on barren spoil top; STS, succession on reclaimed topsoil; PTS, plantation on reclaimed topsoil; BD, bulk density.

### The effect of afforestation on soil structure and water retention

**Fig. 3:**
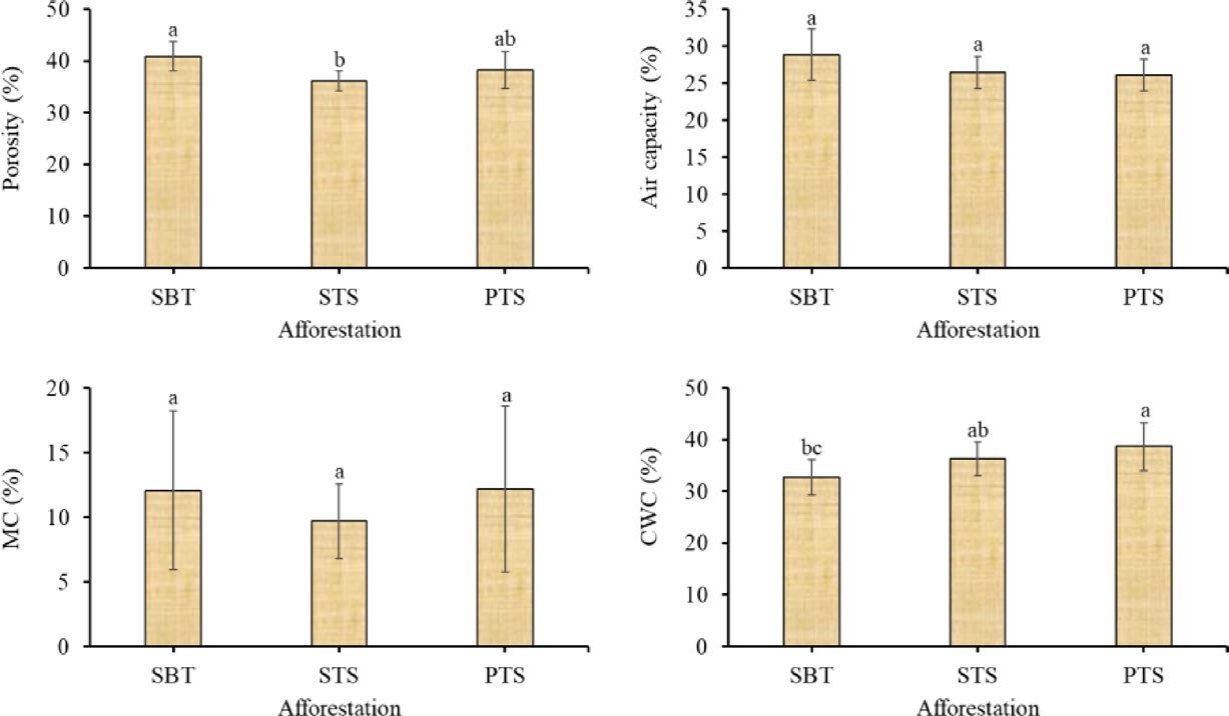
The effect of afforestation on soil structure and water retention. SBT: succession on barren spoil top; STS: succession on reclaimed topsoil; PTS: plantation on reclaimed topsoil; MC: moisture content; CWC: capillary water holding capacity.

Afforestation type significantly affected the soil structure and water retention properties of a post-mining site. The study revealed that porosity was significantly higher under succession on barren spoil top (SBT) than succession on reclaimed topsoil (STS). In contrast, capillary water content (CWC) was significantly higher under plantation on reclaimed topsoil (PTS) than under SBT (Fig. 3).

### The effects of afforestation on soil nutrients and soil quality improvement

Soil nutrients and quality differed significantly among afforestation methods. In particular, Nt, Ca^2^⁺, and K⁺ concentrations were significantly higher in PTS than SBT; conversely, sulfur (St) was significantly higher under SBT than both STS and PTS; Mg^2^⁺ was also higher in SBT than STS (Table 3).

**Table 3:** The effect of afforestation on soil nutrients status.

Soil nutrients	Afforestation		
	SBT	STS	PTS
Nt (%)	0.06 ± 0.03^b1^	0.13 ± 0.06^ab^	0.16 ± 0.06^a^
St (%)	0.29 ± 0.08^a^	0.02 ± 0.00^b^	0.03 ± 0.01^b^
Ca^2+^ (cmol/kg)	0.42 ± 0.04^b^	1.48 ± 0.61^a^	1.50 ± 0.38^a^
K^+^ (cmol/kg)	0.24 ± 0.08^b^	0.36 ± 0.06^ab^	0.43 ± 0.13^a^
Mg^2+^ (cmol/kg)	4.15 ± 0.45^a^	1.88 ± 0.44^b^	3.09 ± 0.88^a^
Na^+^ (cmol/kg)	0.04 ± 0.01^a^	0.03 ± 0.00^a^	0.03 ± 0.01^a^

^1^different letters indicate significant differences at p < 0.05 along the row under different afforestation methods.

### The effect of afforestation on soil organic carbon

**Fig. 4:**
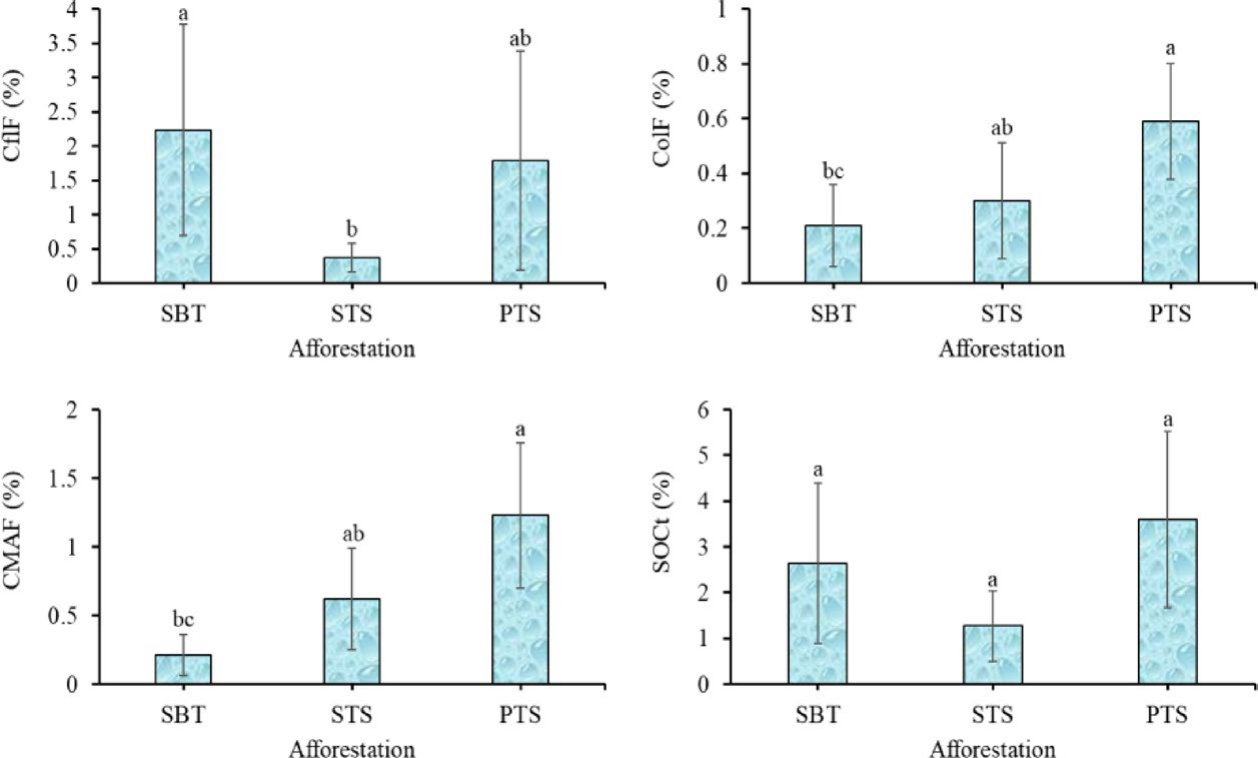
Effect afforestation on soil organic carbon. SBT: succession on barren spoil top; STS: succession on reclaimed topsoil; PTS: plantation on reclaimed topsoil; CflF: free light fraction carbon; ColF: occluded light fraction carbon; CMAF: mineral associated carbon fraction; SOCt: total soil organic carbon.

Soil organic carbon fractions showed significant variability depending on the afforestation types. The free light fraction of carbon (CflF) was found to be higher under SBT than STS. In contrast, both occluded light carbon fraction (ColF) and mineral-associated carbon fraction (CMAF) were significantly higher under PTS than under SBT. However, no significant differences in total soil organic carbon (SOCt) were observed between the afforestation methods (Fig. 4).

### The relationship between soil nutrients and soil carbon fractions

**Fig. 5:**
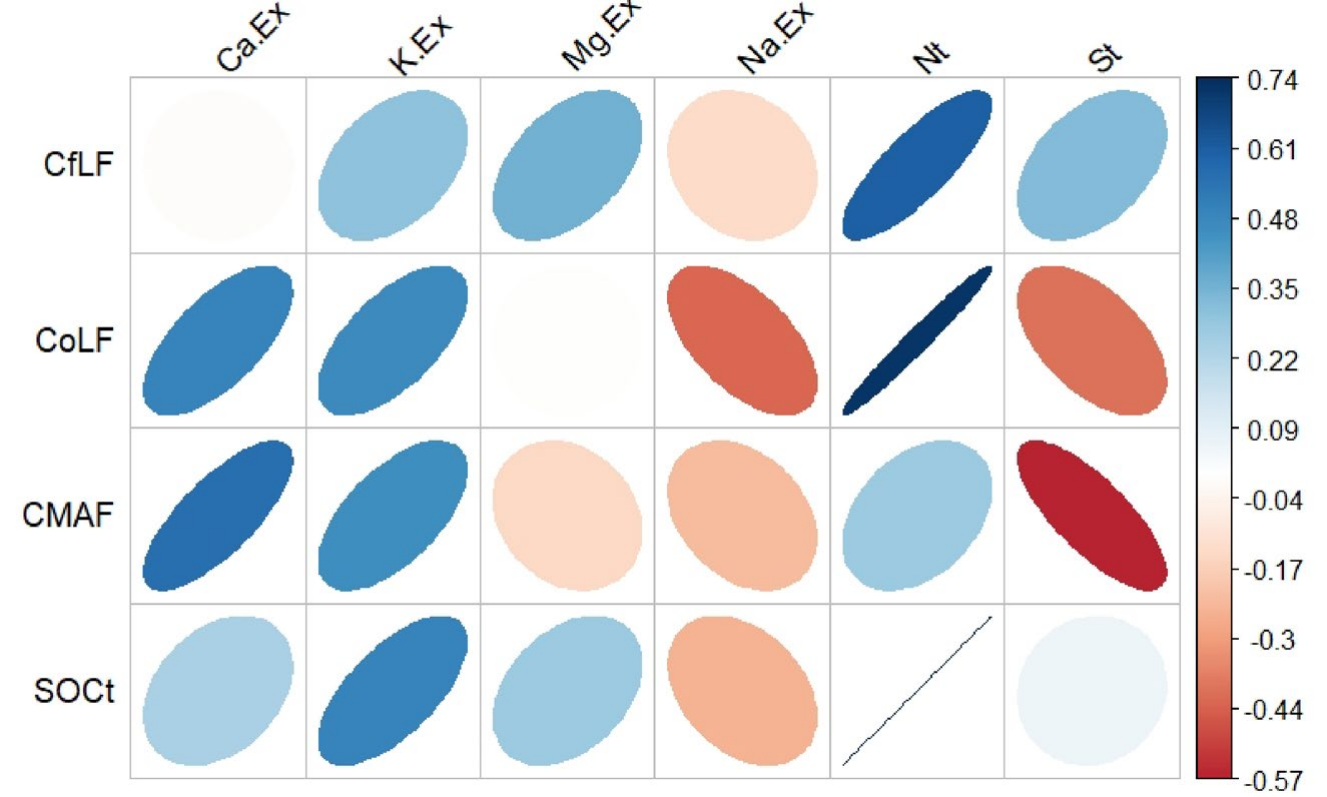
The relationship between soil nutrients and soil carbon fractions.

The free light carbon fraction (CflF) showed a strong positive correlation with total nitrogen (Nt) and a positive correlation with exchangeable potassium (K.Ex), exchangeable magnesium (Mg.Ex) and total sulfur (St). The occluded light carbon fraction (ColF) showed a strong positive correlation with Nt, in addition to positive correlations with exchangeable calcium (Ca.Ex) and K.Ex. In addition, the mineral-associated carbon fraction (CMAF) was positively correlated with Ca.Ex, K.Ex and Nt. Total soil organic carbon (SOCt) showed a strong positive correlation with Nt and positive correlations with Ca.Ex, K.Ex and Mg.Ex (Fig. 5).

## Discussion

### Afforestation effect on soil structure and water retention

Afforestation type significantly influenced the structure and water retention properties of post-mining Technosols. Porosity and capillary water content (CWC) differed significantly among types, whereas air capacity and moisture content (MC) did not vary significantly. The observed higher value of porosity under succession on barren spoil tops (SBT) compared to succession on reclaimed topsoil (STS) can be attributed to compaction caused by heavy machinery during topsoil transportation and grading activities at the reclaimed topsoil site. Such activities are known to compress soil particles, reduce pore space, and increase soil density^39,40^. For example, Shrestha and Lal^41^ reported BD values ranging from 1.55 to 1.86 Mg m^-3^ in soils exposed to heavy machinery during mining and reclamation processes, which is similar to the BD of 1.59 Mg m^-3^ observed under succession on reclaimed topsoil in this study. Furthermore, Čížková et al.^21^ observed that BD was consistently higher in the upper soil layers of reclaimed sites compared to unreclaimed sites, highlighting the influence of mechanical compaction during reclamation activities. However, over a decade, BD of reclaimed sites tends to decrease. For example, Yang and Wang^42^ reported a decrease in bulk density from 1.53 to 1.35 Mg m^-3^ after 10 years of reclamation at a dump site.

In contrast, capillary water content (CWC) is significantly higher in plantations established on reclaimed topsoil (PTS) than on SBT. This difference can be attributed to the improved soil structure and organic matter content associated with reclaimed soils, which enhance their ability to retain water. For example, studies have found that soils with higher organic carbon content have greater water-holding capacity due to increased aggregation and pore size distribution^25,43^. The presence of organic matter not only improves soil structure but also increases the overall water-holding capacity of the soil by creating a network of pores that can retain moisture more effectively^44^.

In addition, the physical properties of soils under different afforestation methods indicate that finer soil particles contribute positively to water retention. Guo et al.^45^ have shown that soils with a higher proportion of fine particles, such as silt and clay, tend to retain more water than coarser textures typically found in barren spoil top (Table 2). This suggests that promoting the development of finer-textured soils and increasing initial soil organic matter through topsoil reclamation practices can significantly improve water retention in post-mining Technosols.

### Effect of afforestation on soil nutrients and soil quality improvement

The enhancement of soil nutrients and overall soil quality varies significantly among different afforestation types in post-mining Technosols. The results indicate that Nt, Ca^2^⁺, and K⁺ concentrations are significantly higher under PTS than under SBT. Various studies have revealed the significant role of topsoil reclamation in the soil nutrients. For instance, a study by Bandyopadhyay et al.^46^ reported that after 25 years of afforestation, Nt stocks in reclaimed soils increased significantly, reaching levels comparable to those in natural forest ecosystems. Misebo et al.^47^ observed 36% higher Nt at mine sites reclaimed with topsoil application than without it. Mukhopadhyay and Masto^48^ reported that reclamation practices resulted in 5.5-fold and 30-fold increases in Nt and K stocks, respectively. These results underscore the important role of reclamation in enhancing soil nutrient stocks through decomposition of organic matter, which is confirmed by the strong correlation of soil organic carbon with Nt, Ca^2^⁺, and K⁺ (Fig. 5).

Total sulfur (St) concentrations were significantly higher in the SBT than both the succession on reclaimed topsoil (STS) and the PTS. This phenomenon can be attributed to the mineralization of sulfur-bearing minerals from barren coal overburden, primarily pyrite (FeS₂), exposed during mining operations. Pyrite oxidation upon exposure to oxygen and water temporarily increases sulfur concentrations in the substrate until vegetation establishment and subsequent biological processes modify soil chemistry^49^. Notably, pyrite is the dominant inorganic sulfur impurity in most coal deposits^50^. In addition, magnesium (Mg^2^⁺) concentrations were found to be higher in the SBT than in the STS. This observation can be attributed to the retention of specific mineralogical characteristics in the barren spoil top that contribute to higher magnesium levels. Ongoing weathering processes in these environments facilitate the release of magnesium ions into the soil solution, thereby increasing their availability^51^. The variation in nutrient availability and soil quality improvements between afforestation methods highlights the critical importance of selecting appropriate reclamation strategies for post-mining landscapes. Consequently, the application of reclaimed topsoil has been shown to support the establishment of productive plant communities that subsequently enhance nutrient cycling.

### Soil organic carbon under different afforestation types

Soil organic carbon (SOC) fractions also exhibit significant variability depending on the afforestation types. The results showed that the free light fraction of carbon (CflF) is higher under SBT than that under STS. This phenomenon may be attributed to the higher sand content under SBT (Table 2) and the proliferation of fine roots in nutrient-deficient soils, where plants increase fine root production to maximize resource acquisition^52^, along with limited microbial colonization for organic matter decomposition caused by the harsh environmental conditions of unreclaimed post-mining sites^53^. This finding suggests that the initial stages of soil development in SBT may favor the accumulation of more readily decomposable organic matter, which is often associated with higher CflF levels. Soil macro- and microorganisms play a vital role in litter decomposition and the stabilization of soil organic matter (SOM) through their feeding activities and the accumulation of microbial biomass and metabolites^54,55^.

In contrast, both the occluded light carbon fraction (ColF) and the mineral-associated carbon fraction (CMAF) were significantly higher under PTS than under SBT. This is likely due to increased humification^56^ and improved soil texture^57^, indicating that reclaimed soils enriched with organic matter and conducive to microbial colonization facilitate carbon stabilization in both ColF and CMAF forms. Similarly, studies by Das and Maiti^58^ and Misebo et al^59^ were found that reclaimed sites exhibited higher levels of stable organic carbon compared to unreclaimed sites at 0–10 cm depth. The stability of organic carbon is influenced more by the intricate interactions among microbial communities, enzymatic activities, substrates, and environmental factors^60,61^.

Despite differences in specific soil organic carbon (SOC) fractions, no significant differences in total soil organic carbon (SOCt) were found among the different afforestation methods. This suggests that while individual SOC fractions may vary, the total SOC stock may remain stable over time under certain conditions, primarily influenced by factors such as the age of the afforested sites and the inherent characteristics of the substrate. This finding contrasts with the observations of Pietrzykowski and Krzaklewski^62^, who found that reclaimed mining soils contained three times more SOC than successional soils. Consequently, the variability of SOC fractions across afforestation practices underscores the complexity of soil carbon dynamics in post-mining environments. A thorough understanding of these dynamics is essential for developing effective land restoration strategies that improve soil health and enhance carbon sequestration potential.

## Conclusion

This study demonstrates that afforestation methods significantly influence soil structure, water retention, nutrient availability, and organic carbon dynamics in post-mining Technosols. SBT showed higher porosity, St, Mg^2^⁺, and CflF, whereas PTS exhibited higher CWC, Nt, Ca^2^⁺, K⁺, ColF, and CMAF—reflecting enhanced soil properties from active reclamation. Importantly, STS showed improvement patterns comparable to PTS for several key properties, indicating that natural succession can also lead to substantial soil recovery. Although SOC fractions varied, total SOC remained consistent across treatments, affirming that both natural succession and active restoration contribute to soil organic carbon accumulation. The choice of afforestation strategy should balance specific reclamation objectives and topsoil availability. As this study was limited to assessing physical and chemical properties, future work should evaluate biological attributes (e.g., microbial diversity) and conduct detailed cost–benefit analyses to further optimize post-mining reclamation strategies.

## Data Availability

Data will be made available on request. If someone wishes to request data from this study, they should contact Amisalu Milkias Misebo, the corresponding author.
